# Exploring the transcriptome of *luxI^−^* and *ΔainS* mutants and the impact of N-3-oxo-hexanoyl-L- and N-3-hydroxy-decanoyl-L-homoserine lactones on biofilm formation in *Aliivibrio salmonicida*

**DOI:** 10.7717/peerj.6845

**Published:** 2019-04-30

**Authors:** Miriam Khider, Hilde Hansen, Erik Hjerde, Jostein A. Johansen, Nils Peder Willassen

**Affiliations:** 1 Norwegian Structural Biology Centre, Department of Chemistry, Faculty of Science and Technology, UiT—The Arctic University of Norway, Tromsø, Norway; 2 Centre for Bioinformatics, Department of Chemistry, Faculty of Science and Technology, UiT—The Arctic University of Norway, Tromsø, Norway

**Keywords:** Quorum sensing, Acyl homoserine lactone, *Aliivibrio salmonicida*, Biofilm, Exopolysccharides

## Abstract

**Background:**

Bacterial communication through quorum sensing (QS) systems has been reported to be important in coordinating several traits such as biofilm formation. In *Aliivibrio salmonicida* two QS systems the LuxI/R and AinS/R, have been shown to be responsible for the production of eight acyl-homoserine lactones (AHLs) in a cell density dependent manner. We have previously demonstrated that inactivation of LitR, the master regulator of the QS system resulted in biofilm formation, similar to the biofilm formed by the AHL deficient mutant *ΔainSluxI^−^*. In this study, we aimed to investigate the global gene expression patterns of *luxI* and *ainS* autoinducer synthases mutants using transcriptomic profiling. In addition, we examined the influence of the different AHLs on biofilm formation.

**Results:**

The transcriptome profiling of *ΔainS* and *luxI^−^* mutants allowed us to identify genes and gene clusters regulated by QS in *A. salmonicida*. Relative to the wild type, the *ΔainS* and *luxI^−^* mutants revealed 29 and 500 differentially expressed genes (DEGs), respectively. The functional analysis demonstrated that the most pronounced DEGs were involved in bacterial motility and chemotaxis, exopolysaccharide production, and surface structures related to adhesion. Inactivation of *luxI*, but not *ainS* genes resulted in wrinkled colony morphology. While inactivation of both genes (*ΔainSluxI^−^*) resulted in strains able to form wrinkled colonies and mushroom structured biofilm. Moreover, when the *ΔainSluxI^−^* mutant was supplemented with N-3-oxo-hexanoyl-L-homoserine lactone (3OC6-HSL) or N-3-hydroxy-decanoyl-L-homoserine lactone (3OHC10-HSL), the biofilm did not develop. We also show that LuxI is needed for motility and for repression of EPS production, where repression of EPS is likely operated through the RpoQ-sigma factor.

**Conclusion:**

These findings imply that the LuxI and AinS autoinducer synthases play a critical role in the regulation of biofilm formation, EPS production, and motility.

## Introduction

Quorum sensing (QS) is a widespread mechanism in bacteria that employs autoinducing chemical signals in response to cell density. A variety of classes of QS chemical signals have been identified in different bacteria. Gram-negative bacteria usually employ N-acyl homoserine lactones (AHLs) which contain a conserved homoserine lactone (HSL) ring and an amide (N)-linked acyl side chain. The acyl groups identified to date range from 4 to 18 carbons in length (Fuqua, Parsek & Greenberg, 2001; Swift et al., 2001; Whitehead et al., 2001). AHL-mediated QS was originally discovered in the marine bacterium *Aliivibrio fischeri*, which was found to regulate bioluminescence through the *lux* operon in a cell-density dependent manner (Nealson & Hastings, 1979). The bacterium *A. fischeri* controls luminescence via the QS systems LuxS/LuxPQ, LuxI/LuxR, and AinS/AinR, where LuxS, LuxI, and AinS are the AHL autoinducer synthases (Lupp & Ruby, 2004, 2005; Lupp et al., 2003).

The marine bacterium *Aliivibrio salmonicida*, is known to cause cold-water vibriosis in Atlantic salmon (*Salmo salar*), rainbow trout (*Oncorhynchus mykiss*), and captive Atlantic cod (*Gadus morhua*) (Egidius et al., 1981, 1986; Holm et al., 1985). The genome sequence of *A. salmonicida* revealed five QS systems of which three are similar to those of *A. fischeri*, the LuxS/PQ, LuxI/R, and AinS/R (Hjerde et al., 2008). In *A. salmonicida* the LuxS/PQ and AinS/R systems transduce the information from the autoinducers AI-2 and 3OHC10-HSL to the histidine phosphotransferase protein LuxU and finally to the response regulator LuxO. The level of phosphorylated LuxO depends on the autoinducer concentrations. The phosphorylated LuxO controls the expression of small regulatory RNAs Qrr that together with the RNA chaperon Hfq, destabilize the transcript of the master regulator LitR.

*A. salmonicida* produces eight AHLs, where the LuxI is responsible for production of seven autoinducers (3OC4-HSL, C4-HSL, 3OC6-HSL, C6-HSL, C8-HSL, 3OC8-HSL, and 3OC10-HSL), and AinS only one autoinducer, 3OHC10-HSL (Hansen et al., 2015). Although, *A. salmonicida* encodes the *lux* operon (*luxCDABEG*) (Nelson et al., 2007), the bacteria is only able to produce bioluminescence after addition of decyl aldehyde (Fidopiastis, Sørum & Ruby, 1999). LitR, the master regulator of QS is a positive regulator of AHL production and hence, cryptic bioluminescence in *A. salmonicida* (Bjelland et al., 2012).

In addition to regulating bioluminescence, AHLs are also involved in several physiological processes in bacteria such as production of virulence factors, drug resistance, motility, and biofilm formation (Abisado et al., 2018; Whitehead et al., 2001). AHL-mediated QS is involved in all stages of biofilm formation from attachment to maturation and dispersal in a number of bacterial species (Emerenini et al., 2015; Fazli et al., 2014; Guvener & McCarter, 2003; Hmelo, 2017; Huber et al., 2001; Pratt & Kolter, 1998; Whitehead et al., 2001; Yildiz & Visick, 2009). In many *Vibrio* species development of biofilm and rugose colony morphology dependent on exopolysaccharide (EPS) production (Yildiz & Visick, 2009). In *A. salmonicida* the EPS is produced by an operon known as the *symbiosis polysaccharide (syp)* operon, which is regulated by LitR via RpoQ sigma factor (Hansen et al., 2014; Khider, Willassen & Hansen, 2018). Mutation in LitR of *A. salmonicida* resulted in strains with wrinkled colonies and three-dimensional biofilm formation (Bjelland et al., 2012). Similar to *A. salmonicida*, inactivation of HapR, the master regulator of *Vibrio cholerae*, resulted in a regulatory state mimicking low cell density (LCD) conditions, where the mutant produced more EPS compared to wild type (Zhu & Mekalanos, 2003). In contrast to the two species mentioned above, *Vibrio parahaemolyticus* and *Vibrio vulnificus*, form biofilm and opaque colonies at high cell density (HCD). The inactivation of the master QS regulators, OpaR and SmcR, resulted in translucent colonies with decreased EPS production (Lee et al., 2013; McCarter, 1998).

We have previously shown that AinS and LuxI in *A. salmonicida* are responsible for the production of eight AHLs that are involved in regulation of biofilm formation (Hansen et al., 2015). When both *luxI* and *ainS* synthases genes were inactivated, no AHL production was observed in *A. salmonicida* and the double mutant (*ΔainSluxI^−^*) produced a biofilm similar to the biofilm of *ΔlitR* mutant (Hansen et al., 2015). In the present work, we aimed to understand the complex regulation of biofilm formation, EPS production and motility using transcriptomic profiling on the *ΔainS* and *luxI^−^* mutants. At HCD, inactivation of *luxI* had a global effect on the transcriptome and resulted in nearly 500 differentially expressed genes (DEGs), whereas deletion of *ainS* only resulted in 29 DEGs under the same conditions. Genes involved in motility and EPS production were among the DEGs in the *luxI^−^* mutant, which may explain the observations that this mutant lacks flagella, is non-motile and produces rugose colonies. The *ΔainS* mutant showed DEGs associated with phosphorylation and was not involved in regulating colony rugosity. Exposing the *ΔainSluxI^−^* double mutant to 3OC6-HSL (LuxI product) or 3OHC10-HSL (AinS product), resulted in restoration of wild type traits and no biofilm formation was observed indicating that both LuxI/R and AinS/R systems are important in the regulation of biofilm formation.

## Materials and Methods

### Bacterial strains and supplements

Bacterial strains used in this study are listed in Table 1. *A. salmonicida* LFI1238 strain and the *A. salmonicida* mutants were grown from a frozen glycerol stock on blood agar base no. 2 (Oxoid, Basingstoke, UK) with a total concentration of 5% blood and 2.5% NaCl (BA2.5) or in Luria Bertani broth (Difco; BD Diagnostics, Berkshire, UK) with a total concentration of 2.5% NaCl (LB2.5).

**Table 1:** Bacterial strains and plasmids used in this study.

Bacterial strains or plasmids	Description	Source
*A. salmonicida*		
LFI1238	Wild type, isolated from Atlantic cod	Hjerde et al. (2008)
*ΔlitR*	LFI1238 containing an in-frame deletion in *litR*	Bjelland et al. (2012)
*ΔainS*	LFI1238 containing an in-frame deletion in *ainS*	Hansen et al. (2015)
*luxI^−^*	LFI1238 containing an insertional disruption in *luxI*, Cm^r^	Hansen et al. (2015)
*ΔainSluxI^−^*	*ΔainS* containing an insertional disruption in *luxI*, Cm^r^	Hansen et al. (2015)
LFI1238-pVSV102	*A. salmonicida* LFI238 carrying pVSV102, Kn^r^	Khider, Willassen & Hansen (2018)
*ΔlitR*-pVSV102	*ΔlitR* carrying pVSV102, Kn^r^	Khider, Willassen & Hansen (2018)
*ΔainS*-pVSV102	*ΔainS* carrying pVSV102, Kn^r^	This study
*luxI^−^*-pVSV102	*luxI^−^* carrying pVSV102, Kn^r^	This study
*ΔainSluxI^−^*-pVSV102	*ΔainS luxI^−^* carrying pVSV102, Kn^r^	This study
*E. coli*		
C118λpir	Helper strain containing pEVS104	Dunn et al. (2006)
DH5αλpir	*E. coli* strain containing GFP plasmid pVSV102	Dunn et al. (2006)
Plasmids		
pVSV102	pES213, constitutive GFP, Kn^r^	Dunn et al. (2006)
pEVS104	R6Korigin, RP4, *oriT, trb tra*, and Kn^r^	Stabb & Ruby (2002)

A seawater-based medium (SWT) used for all assays consists of five g/L of bacto peptone (BD, Biosciences, San Jose, CA, USA), three g/L of yeast extract (Sigma-Aldrich, Darmstadt, Germany), and 28 g/L of a synthetic sea salt (Instant Ocean, Aquarium Systems, Blacksburg, VA, USA).

The green fluorescence protein (GFP) constitutive plasmid pVSV102 and helper plasmid pEVS104 propagated in *Escherichia coli*, DH5αλpir and CC118λpir, respectively.

### Culture conditions

*A. salmonicida* strains were cultivated from single colonies in two ml (LB2.5) at 12 °C, 220 rpm for 2 days (primary culture). The primary cultures were diluted 1:20 and grown at 12 °C, 220 rpm for an additional day (secondary cultures). GFP tagged strains were selected on BA2.5 supplemented with 150 μl/ml kanamycin.

The *E. coli* strains were cultivated in LB or LA (Luria Agar) containing 1% NaCl (LB1 and LA1, respectively) and incubated at 37 °C and 220 rpm.

### Transcriptomics

#### Sample collection

The overnight secondary cultures of *ΔainS*, *luxI^−^*, and *A. salmonicida* LFI1238 wild type were diluted to OD_600_ = 0.05 (optical density measured at 600 nm) in a total volume of 70 ml SWT media supplemented with 2.5% sea salt. The cultures were grown further at 8 °C and 220 rpm in 250 ml baffled flasks. A total of 10 ml of the grown cultures were collected at LCD OD_600_ = 0.30 (early logarithmic phase) and 2.5 ml was collected at HCD OD_600_ = 1.20 (late exponential phase). The collected samples were harvested by centrifugation at 13,000×*g* for 2 min at 4 °C (Heraeus 3XR; Thermo Scientific, Waltham, MA, USA) and preserved in RNA*later* (Invitrogen, Carlsbad, CA, USA). The preserved cultures were stored at −80 °C until RNA extraction.

#### Total RNA isolation and rRNA depletion

The total RNA was extracted from the cell pellets following the standard protocols provided by the manufacturer (Epicenter, Madison, WI, USA). Ribosomal rRNA was removed from the samples using Ribo-Zero rRNA Removal kit for bacteria (Illumina, San Diego, CA, USA) following the manufacturer’s instructions. The quality of total RNA before and after depletion was determined using the Agilent 2100 Bioanalyzer with the RNA 600 Nano and RNA 600 Pico chips, respectively (Agilent Technologies, Santa Clara, CA, USA).

#### RNA sequencing and data analysis

The RNA-sequencing libraries were constructed using the TruSeq Stranded mRNA Library Prep Kit (Illumina, San Diego, CA, USA), and sequenced at the Norwegian Sequencing Center with the Illumina NextSeq 500 system using Mid-Output Kit v2 for a 75-cycle sequencing run.

The sequencing quality of FASTQ files was assessed using FastQC (http://www.bioinformatics.babraham.ac.uk/projects/fastqc). Further analysis of the RNA-Seq data was performed using EDGE-pro v1.0.1 (Magoc, Wood & Salzberg, 2013) and DESeq2 (Love, Huber & Anders, 2014). EDGE-pro was used to align the reads to the *A. salmonicida* LFI1238 genome (Hjerde et al., 2008) and to estimate gene expression levels. Differences in gene expression between wild type and *luxI^−^*, and wild type and *ΔainS* mutants were determined using DESeq2. DESeq2 first estimates size factors for each gene, then estimate the dispersion by fitting this to a model using the negative binomial distribution. Finally, DESeq2 performs a statistical test to see whether there is evidence for the observed differential expression between wild type and mutant genes, which is reported as a *p*-value. Log2 fold changes of the genes were recalculated to x differential expression values (i.e., Δ*ainS*/wt) and genes were defined as significantly DEGs based on a *p*-value ≤ 0.05 and fold change values of ≥2 and ≤−2 equal to log_2_ fold 1 and −1. tRNA and rRNA reads were filtered out before analysis.

PCA plots are automatically generated by DESeq2 and were used for quality control of the biological replicates. DESeq2 normalize differences in gene expression patterns to compute a distance matrix. The *X*- and *Y*-axes in the PCA plot correspond to a mathematical transformation of these distances so that data can be displayed in two dimensions (Love, Huber & Anders, 2014).

The RNA sequence data presented in this study have been deposited in the European Nucleotide Archive (www.ebi.ac.uk/ena) under study accession number PRJEB29457 for *ΔainS* and *luxI^−^* and accession number PRJEB28385 for *A. salmoncida* wild type.

### High-performance liquid chromatography tandem mass spectrometry assay

#### AHL standards

The following AHL standards, purchased from University of Nottingham, UK were: N-3-oxo-butyryl-L-homoserine lactone (3OC4-HSL), N-3-hydroxy-butyryl-L-homoserine lactone (3OHC4-HSL), N-3-hydroxy-hexanoyl-L-homoserine lactone (3OHC6-HSL), N-3-hydroxy-octanoyl-L-homoserine lactone (3OHC8-HSL), N-3-hydroxy-decanoyl-L-homoserine lactone (3OHC10-HSL). In addition, N-butyryl-DL-homoserine lactone (C4-HSL), N-hexanoyl-L-homoserine lactone (C6-HSL), N-3-oxo-hexanoyl-L-homoserine lactone (3OC6-HSL), N-octanoyl-L-homoserine lactone (C8-HSL), N-3-oxo-octanoyl-L-homoserine lactone (3OC8-HSL), N-decanoyl-DL-homoserine lactone (C10-HSL), N-3-oxo-decanoyl-L-homoserine lactone (3OC10-HSL), N-dodecanoyl-DL-homoserine lactone (C12-HSL), N-3-oxo-dodecanoyl-L-homoserine lactone (3OC12-HSL), N-3-hydroxy-dodecanoyl-DL-homoserine lactone (3OHC12-HSL), acetonitrile, and formic acid for HPLC were obtained from Sigma-Aldrich (St. Louis, MO, USA).

#### Preparation of bacterial supernatants for AHL measurements

Two biological replicates were used for all *A. salmonicida* strains. The overnight secondary cultures were diluted to an OD_600_ = 0.05 in a total volume of 60 ml SWT media supplemented with 2.5% sea salt. The cultures were grown further at 8 °C and 220 rpm in 250 ml baffled flasks for 50 h. Cells from one ml were harvested from each culture using 13,000×*g* (Heraeus Fresco 21; Thermo Scientific, Waltham, MA, USA) for 2 min at 4 °C. The supernatants were acidified with 1M HCl before threefold ethyl acetate extraction as previously described (Purohit et al., 2013). The ethyl acetate phase was dried using a rotary vacuum centrifuge (CentriVap; Labconco, Kansas City, MO, USA) for 15 min at 40 °C and then redissolved in 150 μl of 20% acetonitrile containing 0.1% formic acid and 775 nM of the internal standard 3OC12-HSL.

#### Detection of AHL profiles using a mix of HPLC-MS/MS and full scan HR-MS analysis

The detection of AHL was adapted from the methods described previously (Hansen et al., 2015). Briefly, the samples (20 μl) were injected into an Ascentis Express C18 5 cm × 2.1 mm, 2.7 μm reverse phase column (Supelco, Sigma-Adrich, Darmstadt, Germany) using an Accela autosampler (Thermo Scientific, Waltham, MA, USA). The elution was performed using an Accela pump (Thermo Scientific, Waltham, MA, USA) with an acetonitrile gradient in 0.1% formic acid and consisted of 5% acetonitrile for 18 s, followed by a linear gradient up to 90% acetonitrile over 222 s, and finally 90% acetonitrile for 60 s. The column was re-equilibrated for 60 s with 5% acetonitrile in 0.1% formic acid before the next sample was injected. Flow rate was 500 μl/min for all steps.

The separated compounds were ionized in positive ion electrospray using the following settings: sheath gas flow rate 70, auxiliary gas flow rate 10, sweep gas flow rate 10, spray voltage +4.50 kV, capillary temperature 330 °C, capillary voltage 37 V, and tube lens 80 V.

The ionized components where detected using an LTQ Orbitrap XL (Thermo Scientific, Waltham, MA, USA) run in either MS/MS low resolution mode or full scan HRMS mode. C4 AHLs are difficult to detect using full scan HR-MS analysis due to co-eluting isobaric compounds seen in some samples, so these components together with 3OC6 and 3OHC6 where measured using high-performance liquid chromatography tandem mass spectrometry (HPLC-MS/MS) using the LTQ part of the LTQ orbitrap XL. The rest of the compounds were measured using Full Scan HR-MS analysis. The C4’s, 3OHC6, and 3OC6 elute early in the chromatogram and where measured in two segments each with three scan events. Segment 1 ran from 0 to 0.88 min, with the following scan events. m/z 172.10-> (101.2–103.2) (C4-HSL), m/z 186.10-> (101.2–103.2) (3OC4-HSL), and 188.10-> (101.2–103.2) (3OHC4-HSL). Segment 2 ran from 0.88 to 1.76 min with the following scan events: 172.10-> (101.2–102.3) (C4-HSL), 214.10-> (101.2–102.3) (3OC6-HSL), 216.12-> (101.2–102.3) (3OHC6-HSL). Segment 3 ran from 1.76 to 5 min in which the rest of the compounds where measured using only one scan event, FTMS (165–450) resolution 15,000. Target setting was 5 × 10^5^ ions per scan and the maximum injection time was 250 ms. Lock mass was enabled for correction of background ions from caffeine (m/z 195.0877) and diisooctyl phthalate (m/z 391.2843 and m/z 413.2662). The system was calibrated with a mixture of 15 AHLs including the internal standard 3OC12-HSL. The ion chromatograms were analyzed using the Xcalibur v. 2.1.0 software package (Thermo Scientific, Waltham, MA, USA). The mass window was set to eight parts per million. The limit of detection and the limit of quantification for the different AHLs were calculated as previously described (Purohit et al., 2013).

#### Construction of GFP tagged *A. salmonicida* strains

*A. salmonicida* mutants used in this study were constructed previously (Bjelland et al., 2012; Hansen et al., 2015). The *ΔainS* mutant is a complete deletion of the *ainS* gene resulting from a double cross-over event, *luxI^−^* is an insertional mutant constructed by cloning an internal part of the *luxI* gene into a suicide vector pNQ705. The insertional mutant is a result of a single cross-over event and is chloramphenicol resistant, *ΔainSluxI^−^* is the *ΔainS* complete deletion mutant with an insertional mutation in the *luxI* gene using a *luxI*-pNQ705 plasmid (chloramphenicol resistant) and *ΔlitR* is a complete deletion of the *litR* gene using a suicide plasmid and a double cross-over event. All mutants were tagged with GFP using tri-parental mating as described previously (Khider, Willassen & Hansen, 2018). Briefly, the pVSV102 plasmid carrying the gene coding for GFP and kanamycin was transferred from *E. coli* DH5αλpir to the mutant strains using the conjugative helper strain CC118λpir harboring pEVS104 helper plasmid. Donor and helper cells were grown to mid-log phase (OD_600_ = 0.7) in LB1. Recipient strains (*A. salmonicida*) were grown to early stationary phase (OD_600_ = 1.2) in LB2.5. The donor, helper, and recipient strains were harvested (13,000×*g* for 1 min) and washed twice with LB1 before they were mixed in 1:1 ratio and spotted onto BA2.5 plates, followed by overnight incubation at 16 °C. The spotted cells were re-suspended in LB2.5 and incubated for 24 h at 12 °C with agitation (220 rpm). The potential tagged strains were selected on BA2.5 supplemented with 150 μl/ml kanamycin after 5 days. The tagged strains were confirmed using Nikon Eclipse TS100 Inverted Fluorescence Microscope (Nikon, Melville, NY, USA).

### Static biofilm assay

The biofilm assay was performed as described previously (Hansen et al., 2014; Khider, Willassen & Hansen, 2018). Briefly, the overnight secondary cultures were grown to an OD_600_ of 1.3. The secondary cultures were further diluted 1:10 in SWT and a total volume of 300 μl of culture was added to each well of flat-bottomed, non-tissue culture-treated Falcon 24-well plates (BD, Biosciences). For each mutant and the wild type, final concentrations of 1,400 ng/ml of 3OC6-HSL, 100 ng/ml of 3OHC10-HSL, 197 ng/ml of 3OC8, 100 ng/ml of C8, or 400 ng/ml of C6 were added separately to each well. The concentrations of the AHLs were selected based on those *A. salmonicida* produced in “in vitro” experiments (Hansen et al., 2015). The plates were incubated statically at 8 °C, for 72 h and the biofilm was visualized using a Nikon Eclipse TS100 Inverted Fluorescence Microscope (Nikon) at 10× magnification and photographed with Nikon DS-5Mc (Nikon) camera. The biomasses of the biofilms were indirectly quantified using crystal violet. The medium was removed and 300 μl of 0.1% (wt/vol) crystal violet in H_2_O was added. The plates were incubated at room temperature for 30 min. The crystal violet stain was removed by flipping the plates gently. The wells were then washed twice with 0.5 ml of H_2_O. The plates were air dried overnight and the biofilm was dissolved in 0.5 ml of 96% ethanol with agitation (250 rpm) overnight. The dissolved biofilm was diluted 1:10 in 96% ethanol and transferred to a 96-well plate (100 μl/well). The absorbance was measured at 590 nm (Vmax kinetic microplate reader; Molecular Devices, LabX, Midland, ON, Canada).

### Soft agar motility assay

The motility assay was performed using SWT soft agar plates containing 0.25% agar as previously described (Khider, Willassen & Hansen, 2018). Briefly, the secondary overnight cultures were diluted to an OD_600_ of 0.4 and three μl of each culture was spotted onto the soft agar plates and incubated at 8 °C for 5 days. The degree of motility for each strain was monitored every 24 h for 5 days by measuring the diameters of spreading halos on the soft agar plate.

### Colony morphology and adhesion assay

The colony morphology assay was carried out as described previously (Hansen et al., 2014; Khider, Willassen & Hansen, 2018). In short, a 250 μl aliquot was harvested from each secondary overnight culture by centrifugation and the pellet was re-suspended in 250 μl SWT. Two microliters of each culture was then spotted onto SWT agar plates and incubated at 8 °C for 14 days. The colonies were viewed microscopically with Zeiss Primo Vert and photographed with AxioCam ERc5s (Zeiss, Berlin, Germany) at 4× magnification. Adhesion was examined by using the same 14 day old colonies to test their ability to adhere to the SWT agar plates. The assay was performed by touching the colonies using a sterile plastic loop as previously described (Bjelland et al., 2012; Khider, Willassen & Hansen, 2018). The adherence grading was only recorded as “Non adhesive” for smooth and non adherent colonies, “Weak” for slightly adherent and “Strong” for colonies that were impossible to separate from the SWT agar plate.

### Scanning electron microscopy

The overnight secondary cultures of *A. salmonicida* strains were fixed with 2.5% (wt/vol) glutaraldehyde and 4% formaldehyde in PHEM-buffer and incubated for one day at 4 °C. A total of 100 microliters of each sample was mounted on a poly-L-lysine coated coverslip for 5 min. Coverslips were washed three times with PHEM buffer before they were postfixed in 1% (wt/vol) Osmium tetroxide (OsO_4_). Samples were washed an additional three times with PHEM buffer. All samples were dehydrated with a graded series of ethanol solutions at room temperature for 5 min. The samples were dried using hexamethyldisilazane as a drying agent and left to dry in a desiccator overnight before being mounted on aluminum stubs using carbon tape and silver paint. The samples were coated with gold-palladium using a Polaron Range Sputter Coater (Polaron, Quorum Technologies Ltd, East Sussex, UK). Pictures were obtained with Ziess Zigma Scanning Electron Microscopy (Ziess, Berlin, Germany).

All biological assays were carried out in biological triplicates, unless otherwise indicated. The assays were performed in two to three independent experiments to validate the results. The difference between the mutants relative to the wild type in AHL production, biofilm formation, and motility migration zones were calculated using student’s *t*-test. A *p*-value ≤ 0.05 was regarded as significant.

## Results

### AHL profiling of *A. salmonicida* in SWT medium

In our previous studies, AHL profiling of *A. salmonicida* LFI1238 and mutants thereof were performed after growth in LB2.5 medium. However, since SWT medium is required for biofilm formation we first wanted to know whether a change of medium would affect the AHL profiles of *A. salmonicida* wild type and the mutants.

The different *A. salmonicida* strains (LFI1238, *ΔlitR, ΔainS, luxI^−^*, and *ΔainSluxI^−^*) were grown in SWT medium at 8 °C for 50 h (OD_600_ ∼ 2.0) before samples were harvested and analyzed using HPLC-MS/MS. The *A. salmonicida* wild type and mutants showed AHL profiles (Table 2) that were similar to the profiles after growth in LB (Hansen et al., 2015) with the exception of C4 and 3OC4. Thus, the wild type and the *ΔlitR* AHL profiles consisted of six AHLs, where the 3OC6-HSL was the most abundant. No AHLs were detected in the *ΔainSluxI^−^* supernatant. The *luxI^−^* mutant produced only 3OHC10-HSL, and the *ΔainS* mutant produced the remaining five AHLs. Compared to the wild type, the *ΔlitR* mutant produced lower concentrations of the 3OC6-HSL and 3OHC10-HSL confirming that LitR is a positive regulator of these two AHLs also after growth in SWT medium.

**Table 2:** AHL production in *A. salmonicida* LFI1238, *ΔlitR, luxI^−^, ΔainS*, and *ΔainSluxI^−^*.

Strains	3OC6 (nM)	C6 (nM)	3OC8 (nM)	3OC10 (nM)	3OHC10 (nM)	C8 (nM)
LFI1238	8,403 ± 279.3	606 ± 3.5	366 ± 27	67 ± 5.9	161 ± 2.1	28 ± 3.0
*ΔlitR*	5,173 ± 113.6	593 ± 82.3	330 ± 42.1	72 ± 4.7	11 ± 1.70	25 ± 3.4
*luxI^−^*	NF	NF	NF	NF	105 ± 6.7	NF
*ΔainS*	8,691 ± 0.0	709 ± 54.6	382 ± 42.5	89 ± 16.9	NF	30 ± 0.0
*ΔainSluxI^−^*	NF	NF	NF	NF	NF	NF

**Notes:**

The values represent the mean of two biological replicates ± standard deviation.

C4-HSL and 3OC4-HSL were not detected in this analysis.

NF, not found.

### N-acyl homoserine 3OHC10 and 3OC6 downregulate biofilm formation in *A. salmonicida*

**Figure 1:**
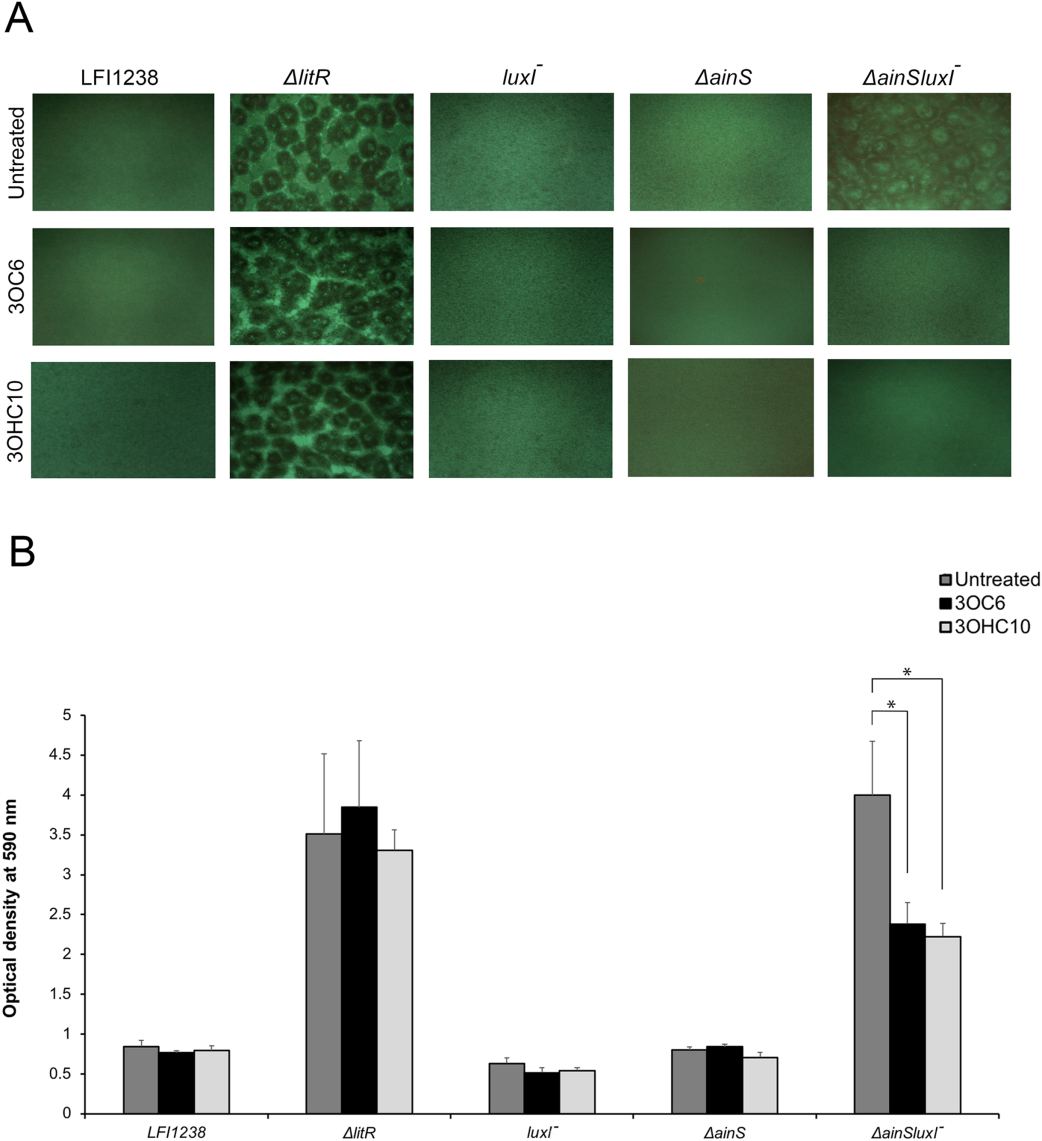
The effect of 3OC6-HSL and 3OHC10-HSL on biofilm formation of LFI1238, *ΔlitR*, *luxI^−^*, *ΔainS*, and *ΔainSluxI^−^*. (A) The strains (LFI1238, *ΔlitR*, *luxI^−^*, *ΔainS*, and *ΔainSluxI^−^*) were allowed to form biofilm in SWT media supplemented with 1,400 ng/ml 3OC6-HSL or 100 ng/ml 3OHC10-HSL at 8 °C for 72 h. The biofilms were viewed in Inverted Fluorescence Microscope Nikon Eclipse TS100 at 10× magnification and photographed with Nikon DS-5Mc camera. (B) The formed biofilms were staining with crystal violet and quantified by measuring the absorbance at 590 nm. The error bars represent the standard deviation of biological triplicates. *Represents *p*-value ≤ 0.05.

Our previously reported results (Hansen et al., 2015) and the AHL profiling presented above, have shown that *litR* deletion significantly influenced the production of 3OC6-HSL (LuxI product) and 3OHC10-HSL (AinS product) compared to the wild type. Therefore, we wished to investigate the possible effects of 3OC6-HSL and 3OHC10-HSL on biofilm formation. The different AHLs were added separately to the SWT medium and *A. salmonicida* strains were allowed to form biofilm at 8 °C for 72 h. As shown in Fig. 1A, the biofilm formation of *ΔainSluxI^−^* was totally inhibited when supplemented with either 3OHC10-HSL or 3OC6-HSL. The *ΔainS, luxI^−^* and the wild type do not form a biofilm (Hansen et al., 2015), and no clear morphological differences in the biofilm formation was observed when treated with 3OHC10-HSL or 3OC6-HSL (Fig. 1A). The mushroom structured *ΔlitR* biofilm remained unchanged after the addition of AHLs. This shows that LuxI-3OC6-HSL and AinS-3OHC10-HSL functions through LitR, and downregulation of biofilm formation cannot be achieved when *litR* is inactivated (Fig. 1A). Next, the biomasses of treated and untreated biofilms were quantified using crystal violet. Relative to the untreated control samples, the addition of either 3OHC10-HSL or 3OC6-HSL significantly decreased the biomass of *ΔainSluxI^−^* biofilm (*p*-value ≤ 0.05). Quantitation of treated and untreated *ΔlitR*, LFI1238, *ΔainS*, and *luxI^−^* showed no significant changes (Fig. 1B). The treatment of *A. salmonicida* wild type and the mutant strains with other AHLs (C6, C8, and 3OC8) did not interfere with the biofilm formation (data not shown). This may indicate that these AHLs are not involved in the regulation of biofilm and have other functions in *A. salmonicida*.

### *luxI^−^* mutant forms adherent wrinkled colonies in *A. salmonicida*

**Figure 2:**
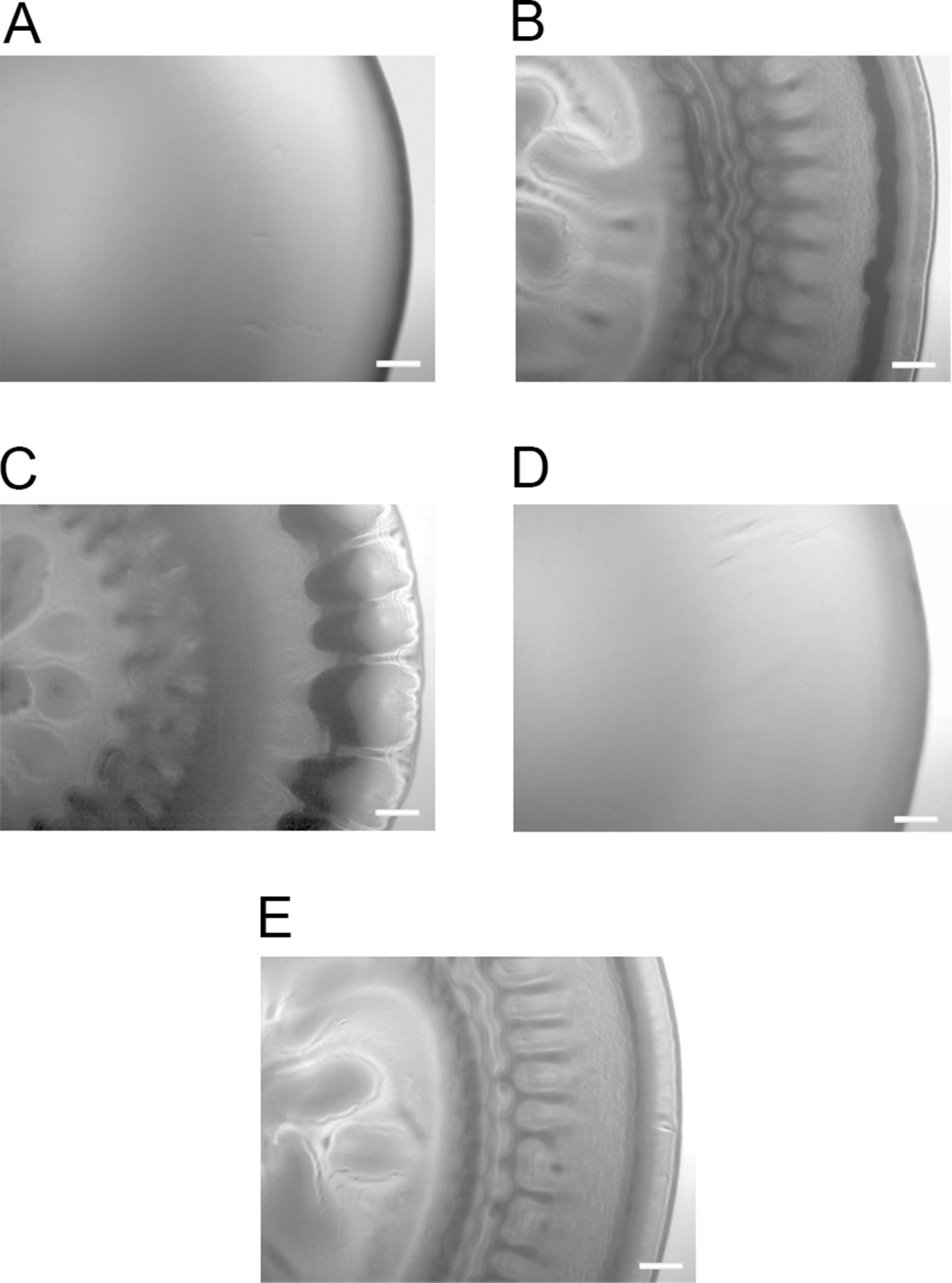
Colony morphology on SWT agar. (A) LFI1238, (B)* ΔlitR*, (C)* luxI^−^*, (D)* ΔainS*, and (E) Δ*ainSluxI^−^*. The colonies of different strains were allowed to form on SWT plates at 8 °C for 14 days. The colonies were viewed in a Zeiss Primo Vert microscope at 4× magnification and photographed with AxioCam ERc5s. Scale bars represent 0.5 mm.

To determine whether any of the *A. salmonicida* QS systems (*lux* and/or *ain*) are involved in the formation of wrinkled colony morphology, the *luxI^−^*, *ΔainS*, and the double mutant *ΔainSluxI^−^* were allowed to form colonies on SWT plates at 8 °C. As shown in Fig. 2, the *ΔainS* mutant formed smooth colonies indistinguishable from those formed by the wild type. This may indicate that *ainS* is not required for formation of rugosity. The *luxI^−^* and *ΔainSluxI^−^* mutants formed wrinkled colonies after 14 days of incubation similar to the colonies formed by *ΔlitR*. The wrinkled colonies formed by the *luxI^−^*, *ΔainSluxI^−^*, and *ΔlitR* mutants were found to be adhesive on the SWT agar at 8 °C. The *ΔainS* mutant behaved similarly to the wild type and produced non-adhesive smooth colonies under the same conditions (Table S1).

### Expression profiles of *A. salmonicida luxI^−^* and *ΔainS* mutants revealed differentially expressed genes related to QS

#### Transcriptome data of A. salmonicida wild type, luxI^−^ and ΔainS mutants

In order to gain a better understanding of the roles of LuxI and AinS work in the QS system, samples from *A. salmonicida* LFI1238 wild type, *luxI^−^* and *ΔainS* mutants were extracted at early logarithmic (OD_600_ = 0.3) and late exponential (OD_600_ = 1.2) phases from three independent cultures. The transcriptome expression profiles (*luxI^−^* and *ΔainS*) of these cultures were compared to the *A. salmonicida* LFI1238 wild type. The total assembled transcriptome of *A. salmonicida* wild type LFI1238 generated an average of 9.87 million reads at LCD and 9.56 million at HCD. The average of mapped reads to the reference genome (*A. salmonicida* LFI1238) was 88.7% at LCD and 91.4% at HCD, with an average mapping coverage of 140.6 and 141.0, respectively. The total assembled transcriptome of *luxI^−^* generated an average of 11.5 million reads at LCD and 9.18 million at HCD. The average of mapped reads to the reference genome (*A. salmonicida* LFI1238) was 94.5% at LCD and 92.9% at HCD, with an average mapping coverage of 109.5 and 85.2, respectively. The detailed transcriptome data of *ΔainS* also showed average of mapped reads to the reference genome of 93.9% and 92.8% at LCD and HCD, respectively, with average mapping coverage of 88 at LCD and 100.8 at HCD (Table S2). To control for technical variations between biological replicates PCA analysis on the expression data from HCD vs. LCD was performed using DESeq2. The biological replicates clustered together well and were distinct between HCD and LCD.

#### The transcriptome profile of A. salmonicida luxI^−^ mutant relative to the wild type

**Figure 3:**
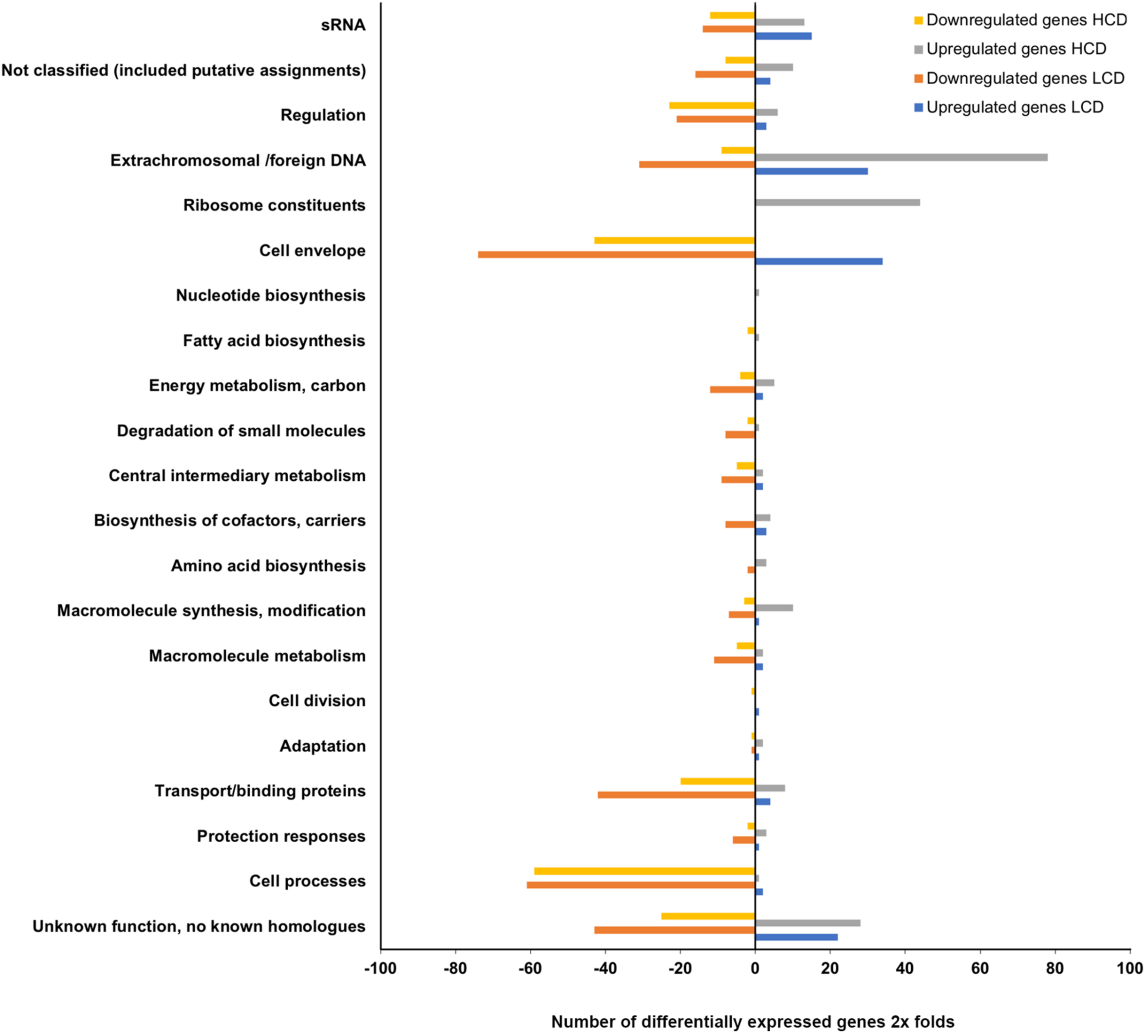
Functional distribution of genes between *A. salmonicida* wild type and *luxI^−^* mutant at HCD and LCD that are ≥2 × differentially expressed. The *x*-axis represents the number of upregulated and downregulated differentially expressed genes of the *luxI^−^*/*wt* at high and low cell densities (filled bars), that fell into various functional groups represented at the *y*-axis.

The expression profiling of *luxI^−^* mutant relative to the wild type LFI1238 revealed 494 and 446 DEGs at LCDs and HCDs, respectively, that fell into various functional gene classes adapted from MultiFun (Serres & Riley, 2000) (Fig. 3). The *luxI^−^* DEGs were distributed almost equally between the large and small *A. salmonicida* chromosomes with 292 DEGs at LCD (59%) and 259 DEGs at HCD (55%). Among the DEGs at LCD 366 were downregulated and 128 were upregulated (Table S3). While at HCD 224 genes were downregulated and 222 genes were upregulated (Table S4). Many of the DEGs we discovered were genes organized in operons. One of the significantly downregulated genes is the *rpoQ* sigma factor (*VSAL_II0319*), organized in an operon of seven genes (*VSAL_II0319–VSAL_II0325*). The whole operon was differentially expressed at HCD and five genes of the operon were differentially expressed at LCD with fold change values ranging from −8.15 to −3.43. The remaining genes of the operon (*VSAL_II0324* and *VSAL_II0325*) were expressed with fold change values −1.74 and −1.93, respectively (Table 3).

**Table 3:** Genes of the *rpoQ*, *tad*, and *syp* operons at low and high cell densities in the *luxI^−^*/wt transcriptome.

VSAL_ID	LCD		HCD		Gene	Function
	FC	*p*-value	FC	*p*-value		
*rpoQ* genes						
*VSAL_II0319*	−8.15	1.78*E*-25	−5.08	9.15*E*-28	*rpoQ*	RNA polymerase sigma factor
*VSAL_II0320*	−5.33	2.33*E*-19	−6.69	6.19*E*-24		Putative membrane associated signaling protein
*VSAL_II0321*	−4.82	1.20*E*-23	−6.54	2.38*E*-32		Putative glycosyl transferase
*VSAL_II0322*	−3.43	3.88*E*-15	−6.52	7.94*E*-33		Putative membrane protein
*VSAL_II0323*	−3.67	1.22*E*-20	−5.67	5.44*E*-24		Putative lipoprotein
*VSAL_II0324*	**−1.74**	0.0001	−4.25	8.65*E*-26		Putative lipoprotein
*VSAL_II0325*	**−1.93**	2.26*E*-05	−4.20	3.29*E*-23		Putative exported protein
*tad* genes						
*VSAL_II0366*	83.84	3.32*E*-45	151.51	2.68*E*-33	*?*	Fimbrial protein. Flp/Fap pilin component
*VSAL_II0367*	57.37	3.31*E*-157	39.53	7.92*E*-39	*tadV*	Type IV leader peptidase
*VSAL_II0368*	22.97	7.63*E*-96	8.23	4.51*E*-32	*rcpC*	Putative Flp pilus assembly protein
*VSAL_II0369*	18.78	5.99*E*-121	10.82	1.54*E*-38	*rcpA*	Type II/III secretion system protein
*VSAL_II0370*	24.63	7.18*E*-155	18.12	4.12*E*-24	*rcpB*	Putative lipoprotein
*VSAL_II0371*	24.11	4.75*E*-138	9.57	1.62*E*-27	*tadZ*	Type II secretion system protein Z
*VSAL_II0372*	20.25	3.92*E*-140	10.26	3.51*E*-33	*tadA*	Type II/IV secretion system protein. ATP binding domain
*VSAL_II0373*	15.02	6.78*E*-77	7.32	2.97*E*-33	*tadB*	Bacterial type II secretion system protein F
*VSAL_II0374*	6.75	4.86*E*-59	2.17	7.26*E*-09	*tadC*	Bacterial type II secretion system protein F
*VSAL_II0375*	3.63	6.47*E*-32	**1.31**	**0.0135**	*tadD*	Putative secretion system protein
*VSAL_II0376*	3.85	3.98*E*-32	**1.23**	**0.1109**	*tadE*	Membrane associated secretion system protein
*VSAL_II0377*	3.97	6.25*E*-43	**1.37**	**0.0301**	*tadF*	Membrane associated secretion system protein
*VSAL_II0378*	3.58	1.99*E*-41	**1.09**	**0.5780**	*tadG*	Membrane associated secretion system protein
*syp* genes						
*VSAL_II0295*			**1.97**	0.001811	***sypR***	Sugar transferase
*VSAL_II0296*			2.49	3.35*E*-09	*sypQ*	Putative transmembrane glycosyl transferase
*VSAL_II0297*			3.63	1.34*E*-15	*sypP*	Putative glycosyl transferase
*VSAL_II0298*			2.13	2.02*E*-07	*sypO*	Putative membrane protein
*VSAL_II0299*			2.04	0.003254	*sypN*	Putative glycosyl transferases
*VSAL_II0300*			3.86	4.63*E*-08	*sypM*	Hypothetical protein
*VSAL_II0301*			**1.07**	0.742157	***sypL***	O-antigen polymerase
*VSAL_II0302*			2.24	8.25*E*-07	*sypK*	Putative polysaccharide biosynthesis protein
*VSAL_II0303*			2.91	3.04*E*-08	*sypJ*	Putative glycosyl transferase
*VSAL_II0304*			3.31	5.28*E*-08	*sypI*	Putative glycosyl transferase
*VSAL_II0305*			**1.01**	0.937840	***sypH***	Putative glycosyl transferase
*VSAL_II0306*			**−1.03**	0.001601	***sypG***	Two-component response regulator. transcriptional regulatory protein LuxO
*VSAL_II0307*			**−1.11**	0.294485	***sypF***	Response regulator. histidine kinase
*VSAL_II0308*			**1.04**	0.691913	***sypE***	Putative response regulator
*VSAL_II0309*			**1.93**	0.001993	***sypD***	Putative capsular polysaccharide synthesis protein
*VSAL_II0310*			3.48	4.60*E*-12	*sypC*	Polysaccharide biosynthesis/export protein
*VSAL_II0311*			6.41	3.45*E*-15	*sypB*	Outer membrane protein. OmpA family
*VSAL_II0312*			9.55	4.70*E*-26	*sypA*	Hypothetical protein. putative anti-sigma factor antagonist

**Note:**

Values indicated in bold are genes filtered out from the analysis due to fold change values (FC) below ≤2 and ≥−2 (not significantly expressed).

**Figure 4:**
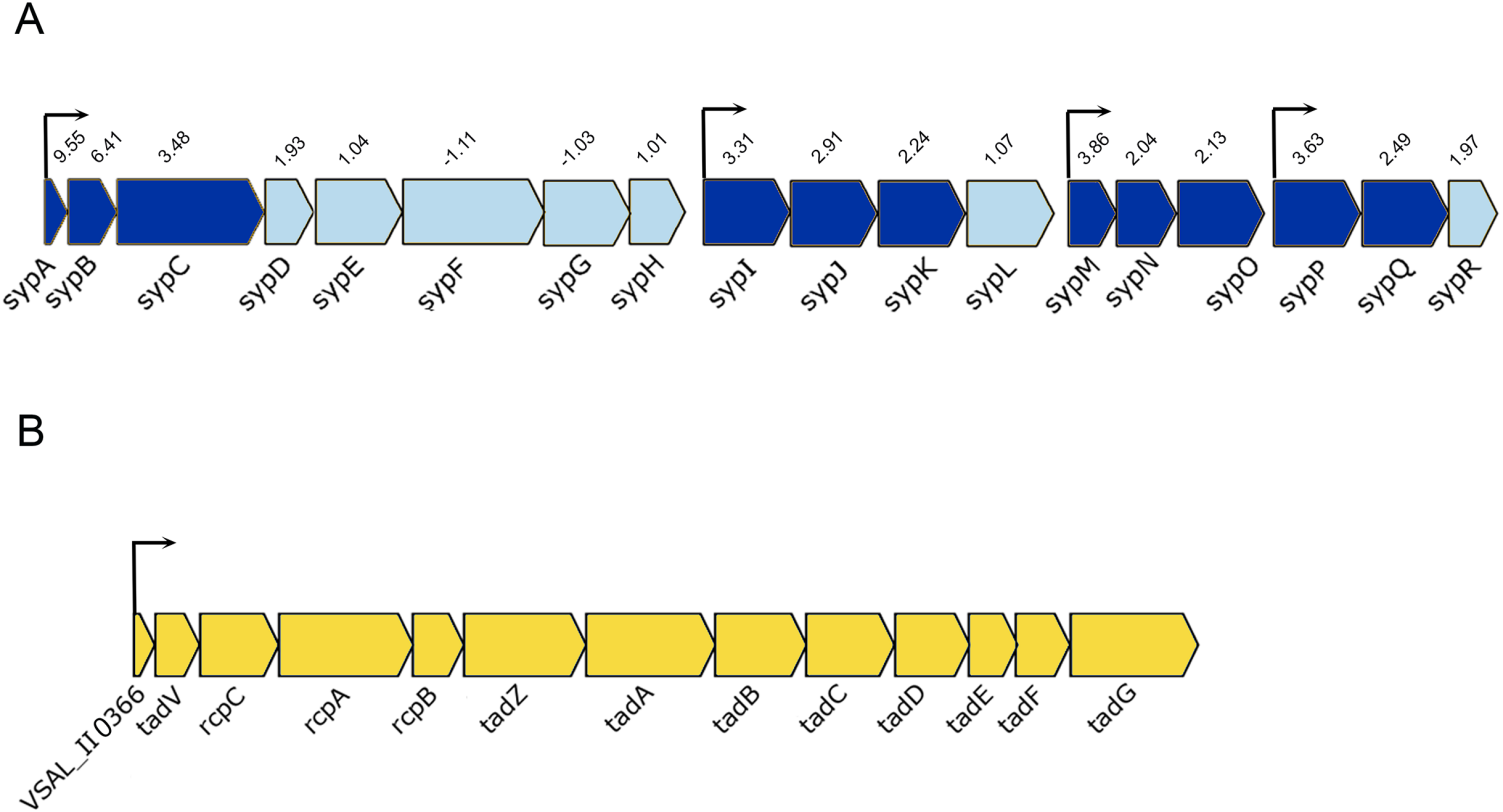
Schematic representation of the genetic organization of *A. salmonicida* *syp* and *tad* operons. (A) The *syp* operon (*VSAL_II0312–VSAL_II0296*) consists of four transcriptional units. Arrows indicate genes and their direction of transcription. The dark blue arrows represent genes with significantly expressed *p*-values, whereas the arrows in light blue are gene with no significant expression values. Number above the arrows indicate the expression level in 2× fold change. (B) The *tad* operon (*VSAL_II0366–VSAL_II0378*), arrows indicate the genes and their direction of transcription.

The *luxI^−^* mutant formed wrinkled colony morphology on SWT plates. The rugosity is associated with the enhanced production of EPSs, which requires the expression of *syp* operon (18 genes) in *A. salmonicida* (Hansen et al., 2014; Khider, Willassen & Hansen, 2018). The *syp* operon (*VSAL_II0295–VSAL_II0312*) is located on chromosome II and organized in four transcription units (Fig. 4A). Our data at HCD demonstrated that 11 genes in the *syp* operon were significantly upregulated with fold change values ranging from 9.55 to 2.04, the remaining genes of the *syp* operon with fold change values ranging from 1.01 to 1.97 were filtered out due to the predominant criteria for identifying DEGs (fold change ≥2 and ≤−2, *p*-value ≤ 0.05) (Table 3).

Among the upregulated genes that fell into the *cell envelope* (*surface structures*) functional group were genes associated with the tight adherence (Tad) loci also known as *tad* operon, that consists of 13 genes located on chromosome II of *A. salmonicida* genome (Fig. 4B). Among the genes of the *tad* operon with highest level of expression were *VSAL_II0366* (83.8-fold change at LCD and 151.5-fold change at HCD) and *VSAL_II0377* (57.3-fold change at LCD and 39.5-fold change at HCD) coding for fimbrial proteins, Flp/Fap pilin component and type IV leader peptidase, respectively. The remaining genes of the *tad* operon were also upregulated in the *luxI^−^* mutant relative to the wild type at both cell densities, and are listed in detail in Table 3. Within the same functional group (*cell envelope*), we were able to identify four downregulated DEGs (*VSAL_I0471*, *VSAL_I0473*, and *VSAL_I0479*) at LCD and one at HCD (*VSAL_I0476*) associated with type IV pilus.

**Figure 5:**
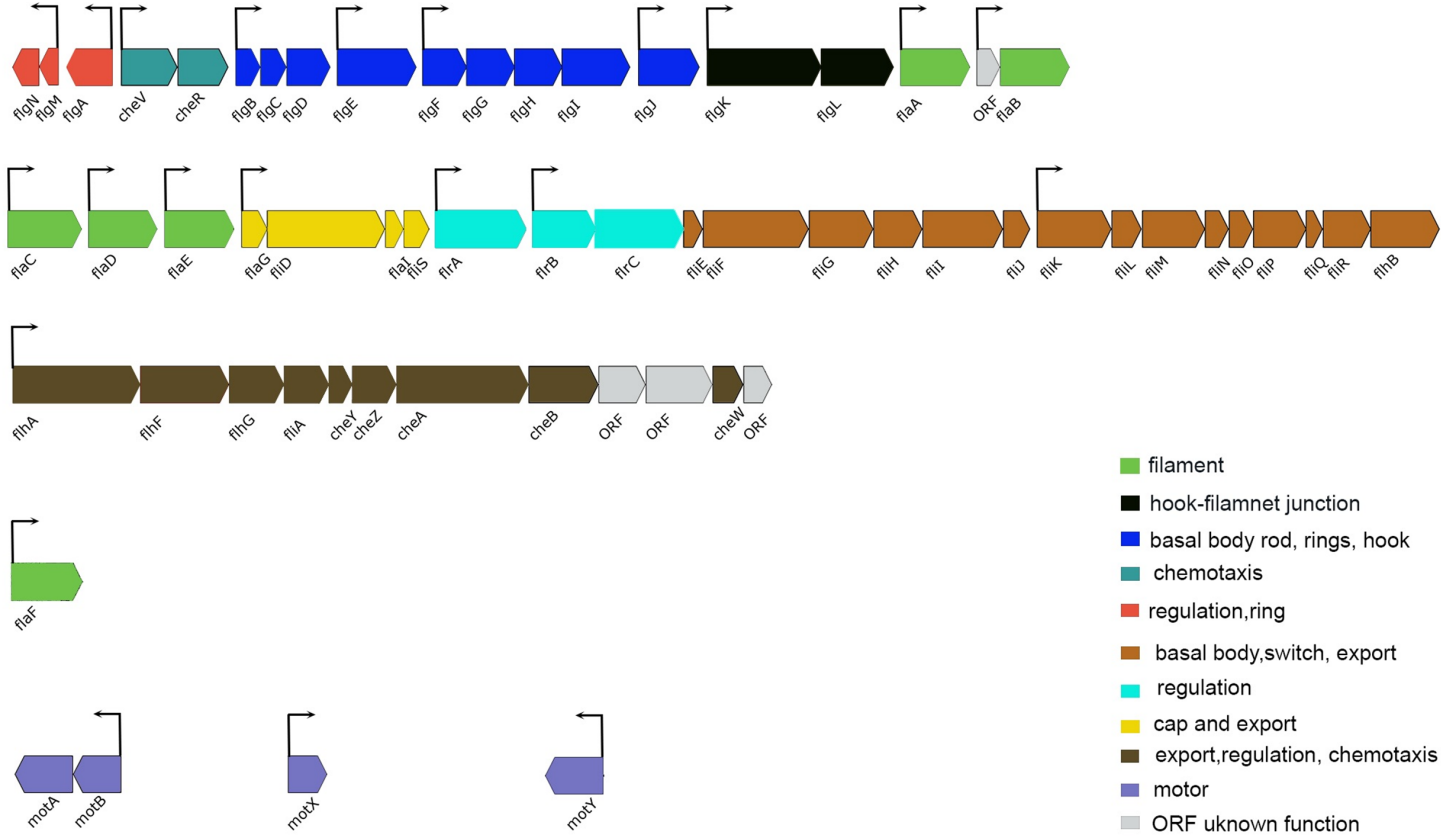
Schematic representation of the genetic organization of *A. salmonicida* flagellar gene system. Flagellar genes of *A. salmonicida* are located in chromosome I and are organized in different chromosomal regions. *flgN* to *flaB* (*VSAL_I2343–VSAL_I2325*) are found in nucleotides (2516889–2499860). *flaC* to *flhB* (*VSAL_I2319–VSAL_I2295*) are found in nucleotides (2492863–2470553). *flhA* to ORF coding for putative membrane protein (*VSAL_I2293–VSAL_I2282*) are found in nucleotides (2466352–2456322). The *flaF* (*VSAL_2517*) is located distinct from the other five filament coding genes (*flaA-flaE*) and is found in nucleotides (2696413–2697570). Motor genes *motA* and *motB* (*VSAL_I0936* and *VSAL_I0937*) are located in an operon and are found in nucleotides (1033690–1035398). *motX* (*VSAL*_*2771*) is found in nucleotides (3000998–3001630) and *motY* (*VSAL_I1863*) is found in nucleotides (1998701–1999579). In *Vibrios* flagellar genes fall into four different hierarchical classes; Class I encode the regulatory protein FlrA, which together with sigma factor 54 controls expression of Class II genes. Class II proteins FlrB and FlrC are important for controlling transcription of Class III genes necessary for synthesis of hook, basal body, and filaments. Class II sigma factor 28 (FliA) regulates transcription of Class IV genes associated with the production of motor components (Brennan et al., 2013; Norsworthy & Visick, 2013; Millikan & Ruby, 2003, 2004; Stewart & McCarter, 2003). Arrows indicate the direction of transcription. The color code provided in the image represent the different functions of each group in the flagellar apparatus.

In several bacteria, QS has been shown to regulate motility and flagellar synthesis (Kim et al., 2007; Ng & Bassler, 2009). The expression profile of the *luxI^−^* mutant revealed genes associated with motility and chemotaxis (59 DEGs at LCD and 57 DEGs at HCD) (Tables S3 and S4). The greatest transcript abundance at LCD and HCD were regulatory genes *flrA* (*VSAL_I2312*) encoding sigma 54-dependent transcription regulator, *flrB* (*VSAL_I2311*) coding for two-component system, sensor histidine kinase and *flrC* (*VSAL_I2310*) coding for response regulator. Other genes coding for flagellin subunits and flagellar basal body rod, ring, hook, and cap proteins, were also downregulated in the *luxI^−^* mutant relative to the wild type at both cell densities. Additionally, genes coding for methyl-accepting chemotaxis proteins and motor proteins as MotA and MotB were downregulated in the *luxI^−^* mutant (Fig. 5).

The transcriptome of *luxI^−^* (*luxI^−^*/wt) showed a downregulation of the *lux* operon, *luxCDABEG* (*VSAL_I0964–VSAL_I0959*). Among the downregulated genes at LCD were *luxC* (−3.53-fold change), *luxD* (−2.09-fold change), *luxA* (−2.14-fold change), and *luxB* (−2.07-fold change) the remaining genes of the operon were not differentially expressed. At HCD the whole operon was significantly downregulated with a fold change values ranging from −51.44 (*luxC*) to −8.21-fold change (*luxG*).

The remaining DEGs of the *luxI^−^*/wt transcriptome were mostly genes with *unknown functions*, *transport/binding proteins, extrachromosomal/foreign DNA*, and *small RNAs* functional groups (Fig. 3).

#### The transcriptome profile of *A. salmonicida* ΔainS relative to the wild type

The transcriptome of *ΔainS* relative to the wild type revealed fewer DEGs compared to the *luxI^−^*. At LCD, a total of 20 DEGs (8 up- and 12 downregulated) were identified. At HCD we were able to identify 29 DEGs where 8 were upregulated and 21 genes were downregulated (Tables S5 and S6). The DEGs fell into 10 functional groups (Fig. S1). At LCD and in the absence of AHLs, the *ain* system of *V. harveyi* act as kinase and serve as phosphoryl-donors to LuxU, which in turn phosphorylates LuxO (Freeman & Bassler, 1999). The *ΔainS* transcriptome demonstrates an upregulation in genes responsible for phosphorylation. The DEGs with significant expression level relative to the wild type was phosphorelay protein LuxU (*VSAL_I1875*) with a fold change values of 2.22 and 2.37 at LCD and HCD, respectively. Whereas the *luxO* gene (*VSAL_I1874*) was not differentially expressed (1.14-fold change at LCD and 1.62-fold change at HCD) and thus not considered further.

Among the upregulated genes that fell into the *surface structures* functional group was the *tad* operon (*VSAL_II0366–VSAL_II0378*). The *VSAL_II0366* gene coding for fimbrial protein showed a fold change values of 2.82 and 4.24 at LCD and HCD, respectively. *VSAL_II0367* coding for Flp/Fap pilin component and type IV leader peptidase was identified among upregulated genes at LCD only (Table S5). Among the 21 genes that were downregulated at HCD, the DEGs with highest fold change values were assigned to *amino acid biosynthesis* functional group including the sulfate adenyltransferase subunit 1 and 2 encoded by *VSAL_I0421* and *VSAL_I0420*, respectively (Table S6).

### LuxI controls motility in *A. salmonicida* LFI1238

The flagellum is required for motility of bacteria, mediating their movements toward favorable environments and avoiding unfavorable conditions (Utada et al., 2014; Zhu, Kojima & Homma, 2013). Since the expression profile demonstrated that a large group of flagellar biosynthesis and assembly genes are regulated by the *lux* system, we wished to analyse the motility behavior of the QS mutants (*luxI^−^*, *ΔainS*, and *ΔainSluxI^−^*), using a soft motility assay.

**Figure 6:**
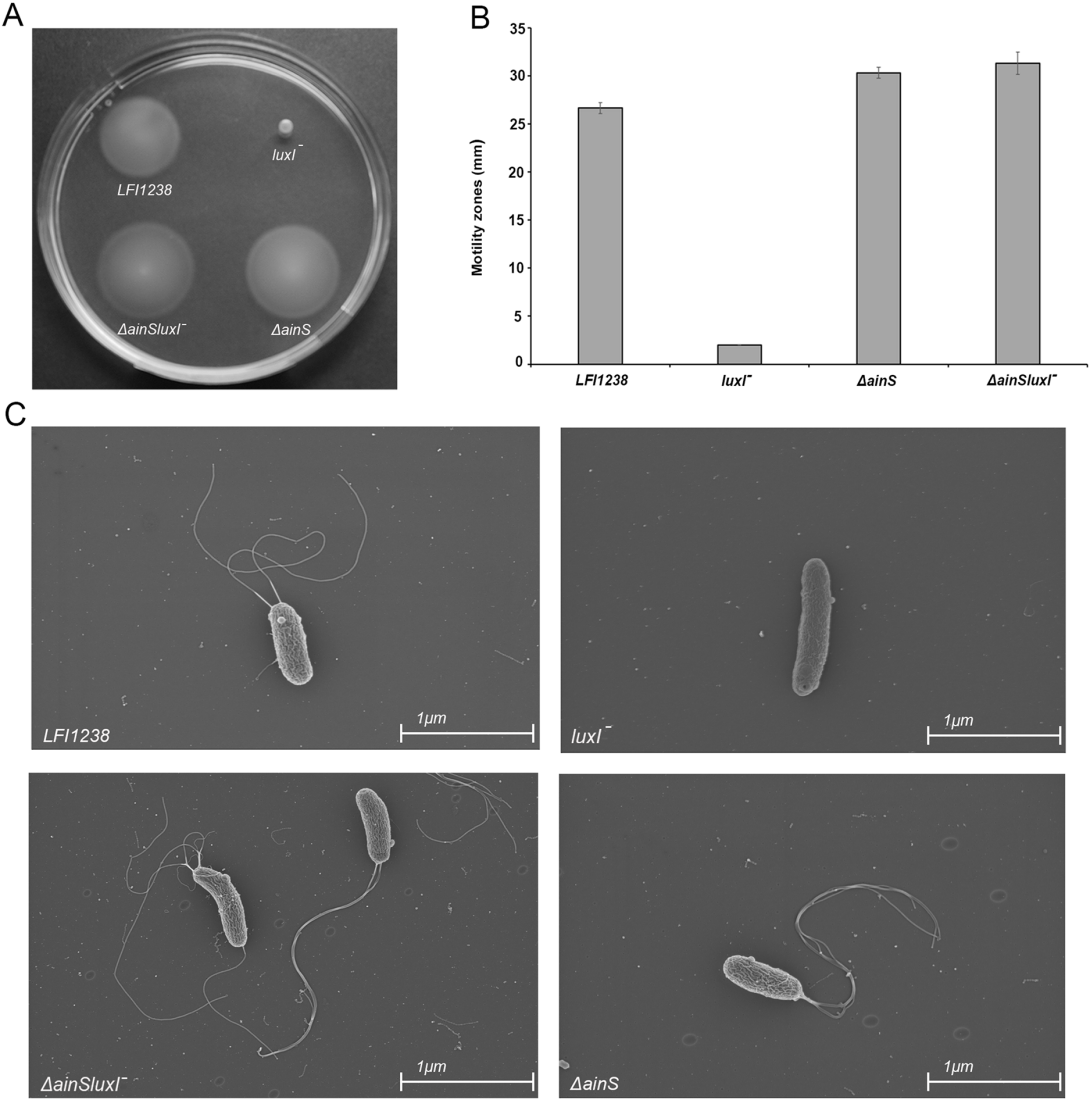
Motility of LFI1238, *luxI^−^*, *ΔainS*, and *ΔainSluxI^−^*. (A) Motility zones on soft agar plates after 5 days on incubation at 8 °C. (B) Measurement of motility zones (mm) of LFI1238, *luxI^−^*, *ΔainS*, and *ΔainSluxI^−^* after 5 days, error bars are standard deviation of biological triplicates. (C) scanning electron microscopy images for flagellum observation of LFI1238, *luxI^−^*, *ΔainS*, and *ΔainSluxI^−^* taken with Ziess Zigma at two kV with an in-lens detector. Scale bars represent one μm.

Our results showed that inactivation of *luxI* resulted in a non-motile strain, where the size of the spotted colony (2.0 mm) did not change, indicating no migration from the site of inoculation (Figs. 6A and 6B). AinS was shown to negatively regulate motility in *A. fischeri* (Lupp & Ruby, 2004), and similarly, we assessed the impact of *ainS* deletion on motility of *A. salmonicida*. Compared to the wild type, which showed motility zones of 26.6 ± 0.57 mm, the *ΔainS* showed an increased motility, where migration through the soft agar resulted in motility zones of 30.3 ± 0.57 mm. Similarly, the *ΔainSluxI^−^* double mutant also demonstrated an increased motility compared to the wild type with motility zones of 31.3 ± 1.15 mm (Table S7). In order to determine whether the strains analyzed by soft motility assay possess or lack flagella, the wild type and the constructed mutants were visualized by scanning electron microscopy. The *ΔainS* and *ΔainSluxI^−^* mutants produced several flagella similar to the wild type. As expected the *luxI^−^* mutant is non-motile and lacks flagella (Fig. 6C).

## Discussion

Acyl-homoserine lactones have been identified in many vibrio and aliivibrio species including *A. salmonicida* (Buchholtz et al., 2006; García-Aljaro et al., 2008; Purohit et al., 2013; Valiente et al., 2009), which showed to produce a broad range of AHLs through LuxI and AinS synthases (Hansen et al., 2015). However, there is still limited understanding of the biological advantages of this AHL diversity in the QS mechanism. In this study, we have demonstrated the influence of *luxI* and *ainS* on the global gene regulation and the impact of AHLs on several phenotypic traits related to QS in order to understand the complex network of signal production and regulation in *A. salmonicida*.

The ability to form rugose colonies and biofilm are often correlated features in vibrios (Casper-Lindley & Yildiz, 2004; Yildiz & Schoolnik, 1999; Yildiz et al., 2004), where wrinkled colony phenotype is generally associated with enhanced EPS production (Yildiz & Schoolnik, 1999). Likewise, in *A. salmonicida* colony wrinkling (rugosity) and biofilm formation requires the expression of *syp* genes responsible for the production of EPS (Hansen et al., 2014; Khider, Willassen & Hansen, 2018). In the study presented here, we show that *luxI^−^* mutant exhibited a strong wrinkling colony morphology, indicating an enhanced polysaccharide production. Earlier studies demonstrated that inactivation of the AHL synthase (*luxI* homologous) in several bacteria caused a reduction in both AHL and EPS production (Koutsoudis et al., 2006; Molina et al., 2005; Von Bodman, Bauer & Coplin, 2003). However, here we show that inactivation of *luxI* in *A. salmonicida* enhanced the EPS production and resulted in wrinkled colonies. Unlike the *luxI^−^* mutants, the *ΔainS* mutant formed smooth colonies, similar to the wild type. These findings are further confirmed by the transcription analysis, which revealed upregulation of 11 *syp* genes and downregulation in the *rpoQ* gene in the *luxI^−^* mutant relative to the wild type. Whereas no DEGs associated with EPS production or *rpoQ* were found in the *ΔainS* transcriptome. The sigma factor RpoQ functions downstream of LitR and is known to be a strong repressor of *syp* in *A. salmonicida* (Khider, Willassen & Hansen, 2018). Although LitR was shown to be a positive regulator of *rpoQ* (Khider, Willassen & Hansen, 2018), the gene (*litR*) was not found among the DEGs of the *luxI^−^* or *ΔainS* transcriptome. We have previously proposed that the expression of *rpoQ* may be independent of LitR and that its regulatory function can be altered by environmental conditions (Khider, Willassen & Hansen, 2018). Hence, our results indicate that the negative regulation of EPS through *rpoQ* is controlled directly or indirectly by LuxI, where this regulation may involve other genes and transcription factors independent of LitR, AinS or the 3OHC10-HSL production. Interestingly, the active AinS and thereby the production of 3OHC10-HSL did not downregulate the rugosity of the *luxI^−^* mutant through the LitR-RpoQ pathway. This can be explained by the late production of the 3OHC10-HSL in *A. salmonicida* (Hansen et al., 2015). We have reported previously that the concentration of 3OHC10-HSL is very low at OD_600_ = 1.2 (the OD_600_ of transcriptomics analysis and morphology assay), which may not be strong enough to induce the LitR-RpoQ cascade involved in the repression of *syp* genes. On the other hand, the *ΔainS* mutant appeared to have wild type colony morphology, indicating a stronger regulatory effect of the LuxI on the RpoQ leading to repression of EPS (via *syp*) and downregulation of rugosity. These results suggest that the *luxI*^−^ mutant is locked into a regulatory state in the bacterial life cycle that requires the EPS production, whereas the *ΔainS* is locked into a different regulatory state, where the EPS production is not necessary.

LitR is suggested to link AinS/R and LuxS/PQ systems to LuxI/R systems in *A. salmonicida*, where its deletion influenced the production of AinS and LuxI AHLs. When both *luxI* and *ainS* were inactivated simultaneously, biofilm and colony morphology similar to *ΔlitR* mutant was formed (Hansen et al., 2015). A simple explanation for this observation is the deficiency in AHL production, leading to *litR* inactivation (Bjelland et al., 2012; Hansen et al., 2014), and thereby no repression of biofilm or colony rugosity is achieved. Furthermore, the exogenous addition of either 3OHC10-HSL (AinS signal) or 3OC6-HSL (LuxI signal) to *ΔainSluxI^−^*, completely inhibited biofilm formation. We have previously shown that the disruption of either EPS or other matrix components (e.g., proteins, lipoproteins, and eDNA), disrupts the mature biofilm formation in *A. salmonicida* (Hansen et al., 2014; Khider, Willassen & Hansen, 2018).

**Figure 7:**
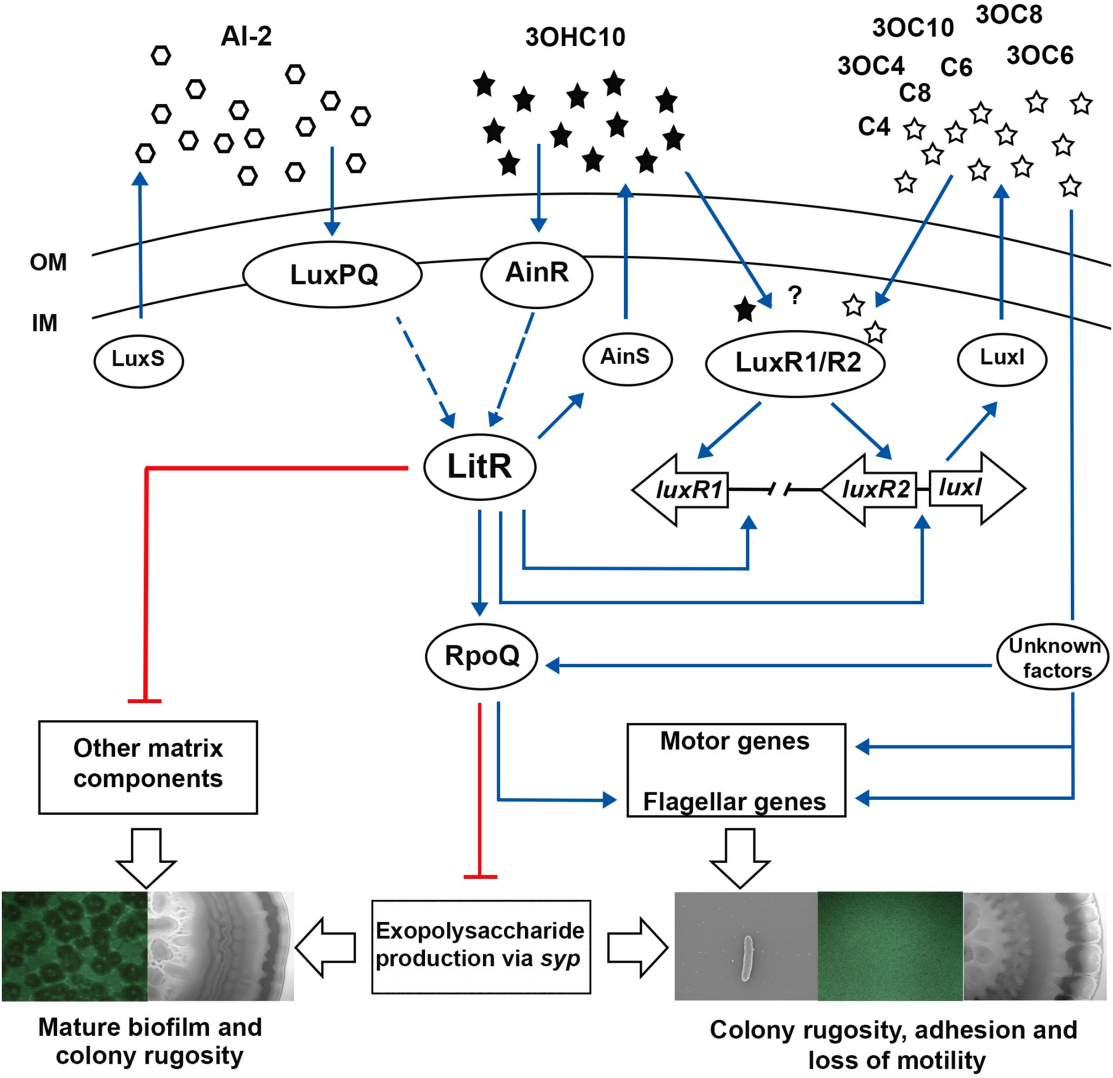
The proposed model of QS system in *A. salmonicida* LFI1238. The autoinducers synthases LuxS, LuxI, and AinS produces AI-2 and eight AHLs that are transported across the outer (OM) and inner membrane (IM) (Hansen et al., 2015). At high cell density AHLs and AI-2 are accumulated to reach a critical concentration to be sensed by their receptors LuxPQ, AinR, or LuxRs. It is still unknown which AHLs bind the LuxRs and is illustrated with a question mark. The Al-2 binds LuxPQ and 3OHC10-HSL binds AinR, which in turn induces a dephosphorylation cascade, resulting in LitR activation. Although due to a frame shift mutation within *luxP*, the LuxS/PQ pathway may not be active. The expressed LitR, activates the production of the AinS AHL (3OHC10-HSL) and the expression of downstream *rpoQ* gene. The increased RpoQ levels represses *syp* operon leading to biofilm disruption and inhibition of colony rugosity. Moreover, LitR represses other matrix components, through a pathway that remain unknown (Khider, Willassen & Hansen 2018). LitR together with LuxRs are proposed to regulate *luxI*. The expressed LuxI mediate the production of seven AHLs and represses *syp* genes via RpoQ either directly or indirectly through unknown factors. Blue arrows and red lines with bar end indicate pathways of positive and negative regulation, respectively, and may consist of several steps. The thicker, empty arrows indicate the resulting phenotypes.

While the *ΔainS* mutant did not produce neither mature biofilm nor wrinkled colonies, introduction of *luxI* mutation to a *ΔainS* background, resulted in strains (*ΔainSluxI^−^*) with three-dimensional biofilm architecture and wrinkled colonies. These data suggest that these two systems regulate biofilm formation synergistically, where the effect of AinS and LuxI AHLs is operated through a common pathway as previously reported (Hansen et al., 2015). The results presented here show that both systems function to either promote or repress production of EPS and other matrix components. However, the *lux* system is believed to be essential for the production of EPS rather than the *ain* system (as discussed above). Studies showed that one key function of EPS involves the attachment of cells to different substratum, which is the initial step in biofilm formation (Vu et al., 2009). For example in *V. cholerae*, the EPS production is the first step in biofilm formation as cells switch from motile planktonic state to being non-motile and surface attached (Silva & Benitez, 2016). Likewise, we suggest that the non-motile *luxI^−^* mutant, increases EPS production to mediate the initial steps in biofilm formation, whereas *ainS* is neither fully activated nor required at this time. This suggests that *lux* system may operate at a lower threshold cell density than *ain* system, which is more essential at later stages of biofilm development, mainly the maturation into three-dimensional mushroom structure. With our results, we expand the previously suggested model, to include *luxI* and *ainS* and their proposed role in regulating biofilm formation and colony rugosity. In the model presented in Fig. 7, we propose that as cell density rises 3OHC10-HSL binds AinR receptor, resulting in activation of LitR, which in turn regulates the production of AinS AHL. The activated LitR leads to a repression on other matrix components required for building a mature biofilm through a mechanism that remain unknown. The activated LitR also leads to increased levels of RpoQ, resulting in repression of EPS through *syp* genes. It has been proposed that *luxI* is activated by both LitR and LuxRs. The active LuxI synthesizes seven AHLs and represses *syp* operon via RpoQ most probably independently of the AinS-LitR pathway. Hence, the production of EPS (via LuxI) and other matrix components (via AinS) appears to play an important role in building the three-dimensional architecture of the biofilm. In summary, our results further support the hypothesis that biofilm formation is a LCD dependent phenotype, when neither LuxI nor AinS AHLs are present. As AHLs accumulate at HCDs, the biofilm is dispersed, indicating that AHL-mediated QS in *A. salmonicida* is involved in the dispersal step of the biofilm cycle. Although further investigations are needed to support this hypothesis.

The deletion of *luxI* was shown to influence expression of 500 genes in the *A. salmonicida*. A similar global regulation of QS regulon has also been observed in *Pseudomonas aeruginosa*, where around 600 genes were believed to be regulated by *las* and *rhI* QS systems (Wagner et al., 2003). The most pronounced regulation in the *luxI*^−^ mutant was observed for genes involved in motility and chemotaxis, exhibiting a significantly low expression level. The regulatory mechanism of motility in *A. salmonicida* remain poorly understood, however, the motility genes are organized in a similar fashion to *A. fischeri* (Karlsen et al., 2008), where flagellar genes are often grouped into different hierarchical classes (Fig. 5). We propose that the loss of motility and flagellation is associated with the elevated level of EPS production by the *luxI*^−^ mutant. Inverse regulation of EPS production and flagellum has been observed in several other microorganisms (Burdman et al., 1998; Garrett, Perlegas & Wozniak, 1999; Prigent-Combaret et al., 1999). In *V. cholerae* O139, strains with inactivated flagellar genes (e.g., *fliK, flhB, fliF, fliE*, and *fliR*) exhibited rugose colony morphology, while mutations in genes coding for motor proteins (*motB* and *motY*) did not display *V. cholerae* strains with a rugose colony morphology (Watnick et al., 2001). We also recently showed that RpoQ in *A. salmonicida* regulates motility and EPS production inversely, where the *ΔrpoQ* mutant exhibited a strong colony rugosity and reduced motility. The transcriptome of *ΔrpoQ* revealed a downregulation in a number of flagellar biosynthesis genes mainly *flaA* (Khider, Willassen & Hansen, 2018; Khider et al., 2019). Our *luxI^−^* transcriptomics results demonstrated DEGs that fell into all hierarchical classes (Fig. 5) including flagellar and motor genes. Thus, it is unclear at which regulatory level LuxI affects motility genes and which motility genes may be associated with the rugose phenotype. Nevertheless, our lack of ability to complement the *luxI^−^* mutant makes it difficult to exclude other factors influencing flagellar synthesis and/or motility and a question remains to be investigated is *whether there is a relationship between the loss of motility and the rugose colony morphology in A. salmonicida*. LitR, is a positive regulator of *ainS* in *A. salmonicida* (Hansen et al., 2015). Thus, not surprisingly, we found that *ΔainS* displayed an increased motility compared to the wild type, similar to that reported for *ΔlitR* (Bjelland et al., 2012). The defect or increase in motility cannot be explained by differences in growth rate, as cultures for different mutants reached the stationary phase at the same rate as the wild type (Fig. S2). However, the regulation of motility in *A. salmonicida* and the target of these regulators still remains to be determined.

Our transcriptome analysis revealed additional genes that are regulated by the *lux* system and might play an important role in adhesion and virulence. Tad loci is a widespread colonization island found in several vibrios and known to play an essential role in motility, biofilm formation, and adhesion (Tomich, Planet & Figurski, 2007). Recent studies in *V. vulnificus* showed a correlation between *tad* genes and the biofilm formation, auto-aggregation, and initiation of attachment to the host (Pu & Rowe-Magnus, 2018). Information regarding the role of *tad* operon in *A. salmonicida* is scant, and the inactivation of *tadV* (*VSAL_II0367*) and *rcpC* (*VSAL_II0368*) did not affect biofilm formation (Hansen et al., 2014). However, our recent transcriptome analysis of the *ΔlitR* and *ΔrpoQ* mutants revealed an upregulation in the *tad* genes, moreover, the functional analysis also showed a strong adhesion of the mutants to the agar plates (Khider, Willassen & Hansen, 2018). Furthermore, the comparison transcriptome of *A. salmonicida* wild type at HCD relative to low, showed a downregulation in *tad* genes at HCD when both LitR and RpoQ were upregulated. This led us to suggest that *tad* genes are essential at LCD and are downregulated by LitR-RpoQ when cell density rises. The *luxI^−^* transcriptome demonstrated an upregulation in *tad* genes with significant fold change values. We have earlier suggested in this work that the *luxI^−^* mutant is locked into the initial steps of biofilm formation, when cells are non-motile, produce high amount of EPS and are adhesive. This suggests further that *tad* genes are important at early stages of the life cycle (e.g., LCD) to mediate attachment and micro-colony formation, which is consistent with previous observations in other bacteria (Nika et al., 2002; Pu & Rowe-Magnus, 2018; Pu et al., 2018; Watnick & Kolter, 1999).

We have previously shown that changes in media composition altered biological traits such as biofilm formation and colony rugosity in *A. salmonicida* (Hansen et al., 2014). Contrary to what was previously reported (Purohit et al., 2013; Hansen et al., 2015) neither C4-HSL nor 3OC4-HSL were detected in the present work, suggesting that the concentration of these AHLs are either below the detectable limit or not produced due to different culturing temperatures and/or media. However, the profiles for the remaining six AHLs were unaffected.

## Conclusion

In this study, we have shown that *luxI*, but not *ainS* is essential for formation of wrinkled colonies at LCD, whereas both systems are required to form a three-dimensional mature biofilm in *A. salmonicida* LFI1238. We also demonstrated that addition of either LuxI-3OC6-HSL or AinS-3OHC10-HSL is able to inhibit biofilm formation. Our results show that *lux* and *ain* systems regulate biofilm formation through a common pathway, where LuxI acts mainly as a repressor of EPS production (*syp* operon) via RpoQ. While AinS is probably involved in the repression of other matrix components required to build the mature biofilm. Furthermore, we identified DEGs associated with motility were regulated by LuxI. These results add a new knowledge to the nature of the QS mechanism of *A. salmonicida*, however further investigations are needed to understand the regulation and complexity of this mechanism.

## Supplemental Information

10.7717/peerj.6845/supp-1 Supplemental Information 1 Functional distribution of genes between *A. salmonicida* wild type and *ΔainS* mutant at HCD compared to LCD that are ≥2× differentially expressed. The number of upregulated and downregulated differentially expressed genes of the *ΔainS/wt* at high and low cell densities (filled bars), that are distributed into various functional groups.

10.7717/peerj.6845/supp-2 Supplemental Information 2 Growth curve of LFI1238, *luxI^−^*, ΔainS, and *ΔainSluxI^−^*. The overnight secondary cultures were diluted to a starting OD_600_ of 0.05 in a total volume of 60 ml SWT. The cultures were grown further in 250 ml baffled flasks at 8 °C and 220 rpm. The optical density was measured every 4 h using Ultrospec 10 cell density meter (Amersham Biosciences). The error bars represent the standard deviation of biological triplicates.

10.7717/peerj.6845/supp-3 Supplemental Information 3 Grading of adherence of LFI1238, *luxI^−^*, *ΔainS*, and *ΔlitR* to SWT agar. The adherence of the colonies was analyzed on SWT agar plates after 14 days of incubation at 8 °C.

10.7717/peerj.6845/supp-4 Supplemental Information 4 Summary of RNA sequencing data.

10.7717/peerj.6845/supp-5 Supplemental Information 5 The differentially expressed genes of *luxI^−^* mutant compared to wild type at LCD.

10.7717/peerj.6845/supp-6 Supplemental Information 6 The differentially expressed genes of *luxI^−^* mutant compared to wild type at HCD.

10.7717/peerj.6845/supp-7 Supplemental Information 7 The differentially expressed genes of *ΔainS* mutant compared to wild type at LCD.

10.7717/peerj.6845/supp-8 Supplemental Information 8 The differentially expressed genes of *ΔainS* mutant compared to wild type at HCD.

10.7717/peerj.6845/supp-9 Supplemental Information 9 Motility zones of LFI1238, *luxI^−^*, ΔainS, and *ΔainSluxI^−^*, formed on soft agar plates. Each value represents the average (mm) of biological triplicates ± standard deviation.

## Abbreviations

AHL: acyl homoserine lactone
GFP: green fluorescent protein
rpm: rounds per minute
RNA: ribonucleic acid
tRNA: transfer RNA
rRNA: ribosomal RNA
h: hours
OD: optical density
RNA-Seq: RNA sequencing
s: seconds
min: minutes

## Additional Information and Declarations

### Data Availability

The following information was supplied regarding data availability: RNA sequencing data are accessible in the European Nucleotide Archive (ENA) under accession numbers PRJEB29457 and PRJEB28385.
