# Supplementary figures and images for: Exploring the transcriptome of luxI− and ΔainS mutants and the impact of N-3-oxo-hexanoyl-L- and N-3-hydroxy-decanoyl-L-homoserine lactones on biofilm formation in Aliivibrio salmonicida
Source: PeerJ. 2019 Apr 30;7:e6845. doi: 10.7717/peerj.6845 (PMC6499059; doi:10.7717/peerj.6845)

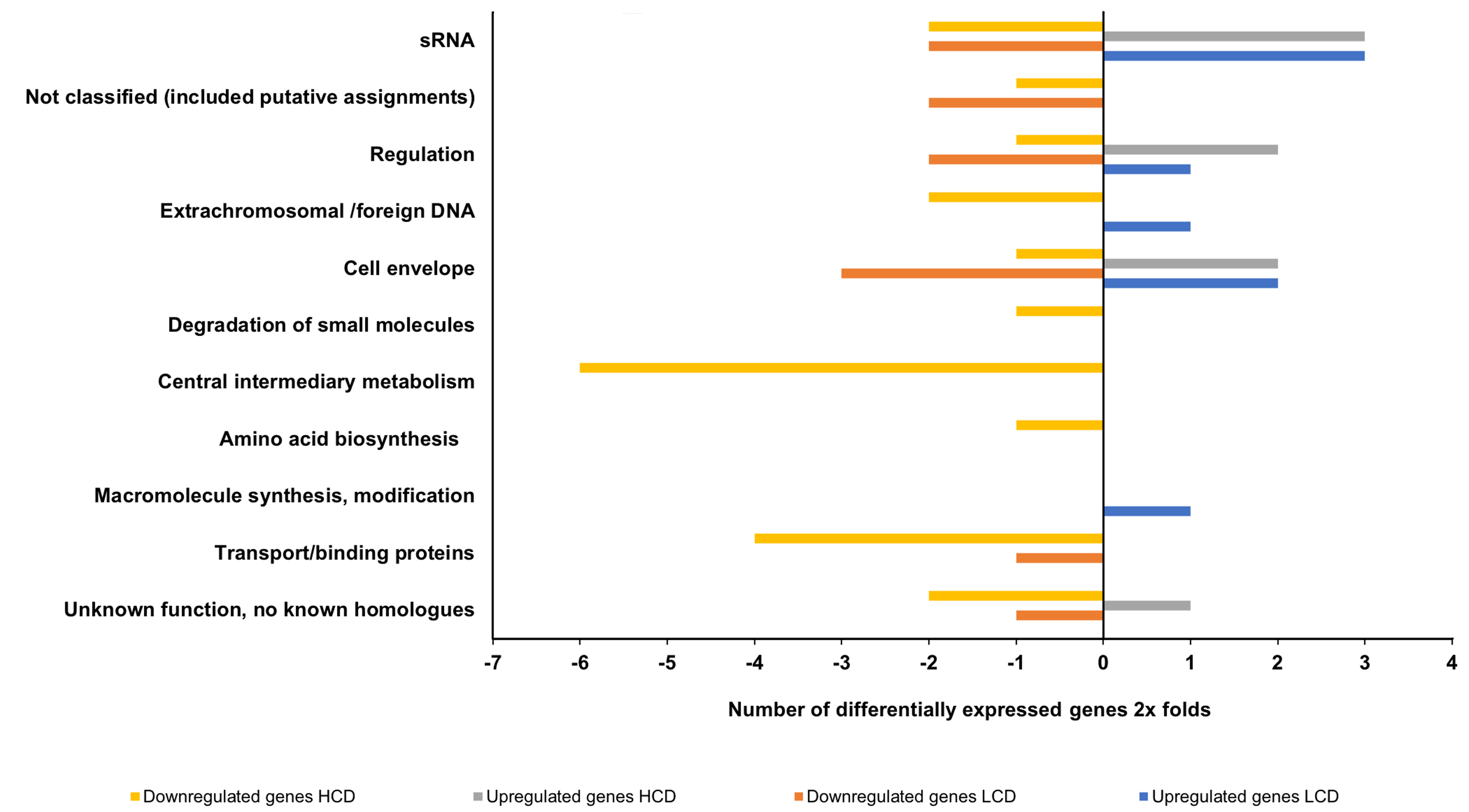

Supplement: Supplemental Information 1 — The number of upregulated and downregulated differentially expressed genes of the ΔainS/wt at high and low cell densities (filled bars), that are distributed into various functional groups. [file peerj-07-6845-s001.png]

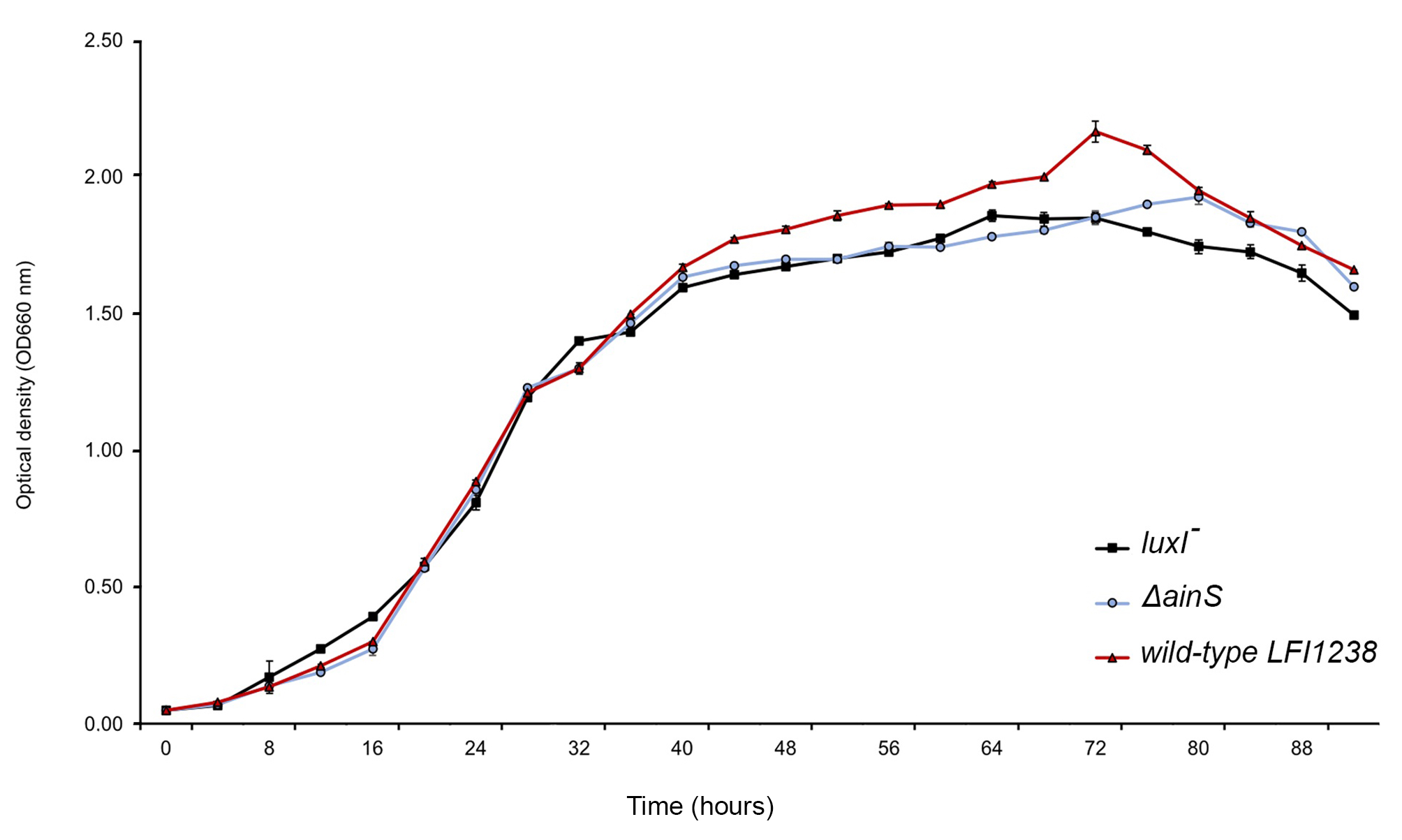

Supplement: Supplemental Information 2 — The overnight secondary cultures were diluted to a starting OD600 of 0.05 in a total volume of 60 ml SWT. The cultures were grown further in 250 ml baffled flasks at 8 °C and 220 rpm. The optical density was measured every 4 h using Ultrospec 10 cell density meter (Amersham Biosciences). The error bars represent the standard deviation of biological triplicates. [file peerj-07-6845-s002.png]
