# Supplementary material for: Exploring the transcriptome of luxI− and ΔainS mutants and the impact of N-3-oxo-hexanoyl-L- and N-3-hydroxy-decanoyl-L-homoserine lactones on biofilm formation in Aliivibrio salmonicida
Source: PeerJ. 2019 Apr 30;7:e6845. doi: 10.7717/peerj.6845 (PMC6499059; doi:10.7717/peerj.6845)
Supplement: Supplemental Information 3 — The adherence of the colonies was analyzed on SWT agar plates after 14 days of incubation at 8 °C. [file peerj-07-6845-s003.docx]

**Table S1**. **Grading of** **adherence of LFI1238, *luxI^-^,*** *Δ****ainS,* and** *Δ****litR*** **to SWT agar.** The adherence of the colonies was analysed on SWT agar plates after 14 days of incubation at 8°C.

| **Bacterial strains** | **8°C** |
| --- | --- |
| LFI1238 | Non adhesive |
| *luxI^-^* | Strong |
| *ΔainS*  *ΔainSluxI^-^* | Non adhesive  Strong |
| *ΔlitR* | Strong |
