# Supplementary material for: Exploring the transcriptome of luxI− and ΔainS mutants and the impact of N-3-oxo-hexanoyl-L- and N-3-hydroxy-decanoyl-L-homoserine lactones on biofilm formation in Aliivibrio salmonicida
Source: PeerJ. 2019 Apr 30;7:e6845. doi: 10.7717/peerj.6845 (PMC6499059; doi:10.7717/peerj.6845)
Supplement: Supplemental Information 9 — Each value represents the average (mm) of biological triplicates ± standard deviation. [file peerj-07-6845-s009.docx]

**Table S7**. **Motility zones of LFI1238, *luxI^-^,*** *Δ****ainS* and** *Δ****ainSluxI^-^*, formed** **on soft agar plates.** Each value represents the average (mm) of biological triplicates ± standard deviation.

| **Bacterial strains** |  |
| --- | --- |
| LFI1238 | 26.6 ± 0.57 |
| *luxI^-^* | 2.0 ± 0.0 |
| *ΔainS* | 30.3 ± 0.57 |
| *ΔainSluxI^-^* | 31.3 ± 1.15 |
|  |  |

* The original size of the spotted colony was 2.0 mm.
